# 3D printed PCLA scaffold with nano‐hydroxyapatite coating doped green tea EGCG promotes bone growth and inhibits multidrug‐resistant bacteria colonization

**DOI:** 10.1111/cpr.13289

**Published:** 2022-07-05

**Authors:** Xiangchun Zhang, Jian He, Liang Qiao, Ziqi Wang, Qinqin Zheng, Chengdong Xiong, Hui Yang, Kainan Li, Chengyin Lu, Sanqiang Li, Hongping Chen, Xulin Hu

**Affiliations:** ^1^ Tea Research Institute, Chinese Academy of Agricultural Sciences Hangzhou China; ^2^ College of Medical, Henan University of Science and Technology Luoyang China; ^3^ The First Affiliated Hospital College of Clinical Medicine of Henan University of Science and Technology Luoyang People's Republic of China; ^4^ Chengdu Institute of Organic Chemistry, Chinese Academy of Sciences Chengdu Sichuan China; ^5^ State Key Laboratory of Oral Diseases and National Clinical Research Center for Oral Diseases and West China Hospital of Stomatology Sichuan University Chengdu China; ^6^ Clinical Medical College and Affiliated Hospital of Chengdu University, Chengdu University Chengdu China

## Abstract

**Objectives:**

3D‐printing scaffold with specifically customized and biomimetic structures gained significant recent attention in tissue engineering for the regeneration of damaged bone tissues. However, constructed scaffolds that simultaneously promote bone regeneration and in situ inhibit bacterial proliferation remains a great challenge. This study aimed to design a bone repair scaffold with in situ antibacterial functions.

**Materials and Methods:**

Herein, a general strategy is developed by using epigallocatechin‐3‐gallate (EGCG), a major green tea polyphenol, firmly anchored in the nano‐hydroxyapatite (HA) and coating the 3D printed polymerization of caprolactone and lactide (PCLA) scaffold. Then, we evaluated the stability, mechanical properties, water absorption, biocompatibility, and in vitro antibacterial and osteocyte inductive ability of the scaffolds.

**Results:**

The coated scaffold exhibit excellent activity in simultaneously stimulating osteogenic differentiation and in situ resisting methicillin‐resistant Staphylococcus aureus colonization in a bone repair environment without antibiotics. Meanwhile, the prepared 3D scaffold has certain mechanical properties (39.3 ± 3.2 MPa), and the applied coating provides the scaffold with remarkable cell adhesion and osteogenic conductivity.

**Conclusion:**

This study demonstrates that EGCG self‐assembled HA coating on PCLA surface could effectively enhance the scaffold's water absorption, osteogenic induction, and antibacterial properties in situ. It provides a new strategy to construct superior performance 3D printed scaffold to promote bone tissue regeneration and combat postoperative infection in situ.

## Graphical abstract

Schematic diagram of the 3D polymerization of caprolactone and lactide (PCLA) coated scaffold containing epigallocatechin‐3‐gallate (EGCG)‐modified nano‐HA as an artificial bone matrix with biphasic function to efficiently promote the growth of osteoblasts and inhibit methicillin‐resistant *Staphylococcus aureus* colonization in the bone repair microenvironment. PCLA/KH‐HA‐EGCG exhibited satisfactory antibacterial properties and leads to significant osteoinduction and osteogenic differentiation in osteoblasts cells, achieving a high‐efficient bone repair effect.

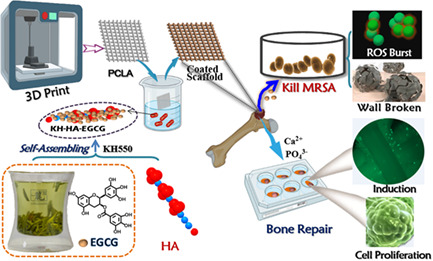

## 1 INTRODUCTION

3D bioprinting technique has been widely used to manufacture tissues and organoids, such as skin, bones and blood vessels, which had drawn much attention in regenerative medicine.^1^, ^2^, ^3^, ^4^, ^5^, ^6^, ^7^, ^8^ However, the application of 3D bioprinting scaffolds in tissue engineering has usually been hampered because scaffolds possess a smooth surface that limits cell adhesion, and its printing process usually requires the assistance of high temperature or organic solvent, making it difficult to integrate bioactive molecules on the scaffold during the printing.^9^ To address this issue, many polymer or natural materials have been developed for fabricating scaffolds to increase their potential for biomedical applications.^10^, ^11^ High molecular polymer materials were considered as bone substitutes owing to their stiffness could meet the mechanical properties of the implantation site, helping to regenerate damaged areas. However, they had several inherent deficiencies when used as fillers in vivo, including low biological activity, poor osteoconductivity and inducibility, and possible immune responses as foreign objects.^12^, ^13^ Therefore, the design and construct a multifunctional 3D printed scaffold for bone repair and bone regeneration is urgently needed.

Currently, several methods are under study to endow bone repair scaffolds with bone regeneration properties, such as modifying the composition of biomaterials and using specific growth factors to stimulate cell adhesion and guide new tissue formation.^14^, ^15^ Among these approaches, some multiphase scaffolds with proper mechanical capabilities were manufactured for bone regeneration and bone repair.^16^ However, few of these scaffolds could simultaneously achieve bone regeneration and in situ anti‐infection.^17^, ^18^ It is reported that 10% of biomaterial‐mediated bone repair was accompanied by bacterial infection after surgery,^19^ such as osteomyelitis caused by *Staphylococcus aureus*. Not to mention the formation of bacterial biofilms on the scaffold surface, where colonizing bacteria is prone to develop resistance to antibiotics.^20^ Clinically, patients are treated with antibiotics after bone repair surgery to prevent or treat bacterial infections at the site of the bone defect. However, the overuse of antibiotics will not only cause physical damage to patients, but also trigger an outbreak of multidrug resistant bacteria, posing a serious threat to human health worldwide.^21^, ^22^, ^23^ Hence, a coating with dual biological activities of antibacterial and osteogenesis, combined with high‐performance polymer to repair bone defects, is urgently desired.

An ideal strategy is to use natural bioactive molecules to integrate into scaffolds, giving them multifunctional properties.^24^ Polymerization of caprolactone and lactide (PCLA) is a macromolecular copolymer prepared by bulk ring‐opening polymerization of caprolactone (PC) and lactide (LA) monomers and has been exploited for medical application via controllable temperature‐sensitive hydrogel or drug delivery capsules.^25^, ^26^ The degradation rate of the copolymer could be controlled by the monomer and reduces the acidity of the degradation products, which is an ideal scaffold for bone support and cell adhesion. The acicular nano‐hydroxyapatite (HA) can increase additives' biomineralization and cell proliferation ability.^27^ If HA is integrated into the PCLA scaffold, it will confer multifunctional properties to the scaffold. We previously reported that the ethoxy group of the silane coupling agent was hydrolyzed to form a silanol reaction with the hydroxyl group of HA, resulting in a silicon‐oxygen bond formation, and thereby enhancing the interface compatibility between HA and polymers, making it possible to modify HA onto PCLA.^28^ As the first botanical prescription drug approved by the FDA, Veregen (PolyphenonE, a green tea extract) is used to treat genital warts caused by human papillomavirus infection. Epigallocatechin‐3‐gallate (EGCG) is the most biologically active and abundant polyphenolic compound in green tea, which possesses antibacterial, antiviral, anti‐inflammatory, antitumor and immune‐modulating activities.^29^ Therefore, we hypothesize that using a silane coupling agent (KH550) as a linking agent to modify HA with EGCG and integrated it into PCLA may be applied for the regeneration of bone defects by relying on the advantages of EGCG and hydroxyapatite.

**FIGURE 1:**
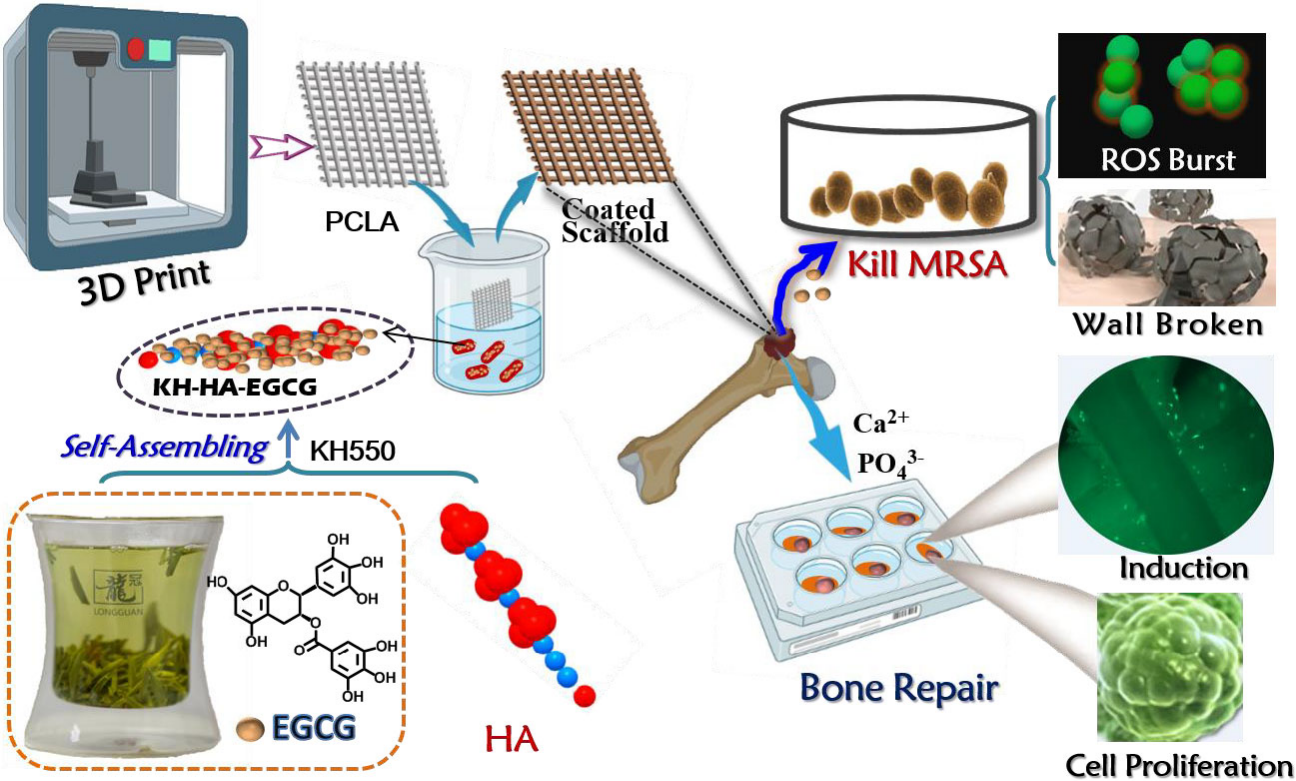
Schematic diagram of the 3D PCLA coated scaffold containing EGCG‐modified nano‐hydroxyapatite (HA) as an artificial bone matrix with biphasic function to efficiently promote the growth of osteoblasts and inhibit MRSA colonization in bone repair microenvironment. PCLA/KH‐HA‐EGCG exhibited satisfactory antibacterial properties and leads to significant osteoinduction and osteogenic differentiation in osteoblasts cells, achieving a high‐efficient bone repair effect. EGCG, epigallocatechin‐3‐gallate; MRSA, methicillin‐resistant *Staphylococcus aureus*; PCLA, polymerization of caprolactone and lactide

In this study, a PCLA scaffold was manufactured by using polyvinyl alcohol as the 3D printing to imitate the periosteum. Then, a general method was developed by using EGCG self‐assembled anchored in the HA with KH and coating the PCLA scaffolds (PCLA/KH‐HA‐EGCG). Taking advantage of the combined effect of EGCG and HA, the PCLA/KH‐HA‐EGCG scaffold had the following functions: (1) distinguished mechanical properties for bone support; (2) significantly trigging osteoinduction and osteogenic differentiation in osteoblasts cells by releasing Ca^2+^ and PO_4_^3−^; (3) in situ killing of methicillin‐resistant *Staphylococcus aureus* (MRSA) by causing reactive oxygen species (ROS) burst and disrupting bacterial wall structure; and (4) exhibiting excellent biocompatibility and biosafety. This scaffold construction strategy that simultaneously promotes bone repair and inhibits bacterial colonization in situ has great significance for treating bone injury, especially bacterial infections in the early bone defect process (Figure 1).

## 2 MATERIALS AND METHODS

### 2.1 Materials

PCLA was synthesized by ring‐opening the bulk polymerization of caprolactone (CL) and lactide (LA), provided by Chengdu Institute of Organic Chemistry, Chinese Academy of Sciences (Viscosity: 2.2‐1.4). The acicular nano‐hydroxyapatite (HA), and Epigallocatechin gallate (EGCG) were purchased from Shanghai Macklin Biochemical Co., Ltd. Acetic acid and polyvinyl alcohol (PVA, 101–110 KDa, 87%–89% hydrolyzed) were obtained from Maya Reagent Co., China. Silane Coupling Agent (KH550) was purchased from Beijing bainuowei biology science and technology Co., ltd. CCK‐8 reagent (Fdbio science), Calcein/PI Live/Dead Viability Assay Kit, crystal violet and Calcium Colorimetric Assay Kit were purchased from Beyotime Biotechnology (China). MC3T3‐E1, HepG2 and A549 cell lines were acquired from ATCC. Cell culture medium and fetal bovine serum (FBS) were supplied from HyClone (USA). Ultrapure water (18 MΩ) was used throughout the study.

### 2.2 Fabrication of HA‐EGCG and KH‐HA‐EGCG nano‐particles

In the typical experiment, 4.0 ml EGCG (25 mg/ml in phosphate buffered saline [PBS]) was mixed with 26 ml of 1 g HA at 40°C in 75% ethanol under vigorous stirring. After 1 h, 1.0 ml of KH550 was introduced in the sample and the PH of mixture solution was adjusted using 2 mM NaOH aqueous solution into 10–12 for another 8 h to produce KH‐HA‐EGCG. HA‐EGCG was prepared by a similar procedure without KH550. Furthermore, the as‐fabricated nano‐particles were filtered to cut off free EGCG and KH550 by a molecular sieve (Millipore, MWCO: 3 K). The obtained nano‐particles are resuspension and stable in 75% ethanol for a long storage period at 4°C.

### 2.3 Fabrication of PCLA/KH‐EGCG scaffolds

PCLA polymer was spurted out layer and layer at an extrusion speed of 15 mm/s at 200 kpa by a 3D printer (EvisionTec, Beijing, China) with a 200 μm diameter nozzle to form a single‐layer cubic porous scaffold with a thickness of 200 μm. In addition, the filling rate was 35%, and the printing was performed under the conditions of 25°C at room temperature and 0°C on an ice plate. Printing conditions: nozzle temprature, 190°C, moving speed, 10 mm/s, pressure 200 kPa. First, PCLA scaffolds were immersed in ethanol solvent. Next, PCLA scaffolds were shaken slowly for 4 h to remove residual solvent and impurities on the surface. Then, samples were washed with deionized water and dried in a vacuum. Meanwhile, the inorganic particles (1 ml) dispersed in alcohol were centrifuged to remove the supernatant and resuspend them in a mixed solution of PVA (0.5%) and acetic acid (vol/vol, 1:1) to form a coating solution. Next, the as‐prepared PCLA scaffolds were immersed in coating aqueous solution for 30 min, vacuum drying, PBS cleaning, and so completed one coating process. After finishing the five coating processes, PCLA/KH‐HA‐EGCG scaffolds were obtained. PCLA/KH‐HA and PCLA/HA‐EGCG scaffolds were acquired by a similar procedure with different substances as a coating.

### 2.4 Characterization of the 3D porous scaffold

The surface morphology of the scaffolds was characterized using scanning electron microscopy (SEM, Phenom Pro, Netherlands). The functional groups were identified by Fourier‐transform infrared spectroscopy (FTIR; Thermo Fisher, Nicolet™ iS™ 10, Shanghai, China). Before performing FTIR, the nanoparticle sample must be vacuum dried first and then mixed with KBr powder to form a thin sheet for testing. The average particle sizes of EGCG modified HA were analysed by a particle size analyser (Mastersizer2000, China). The water uptake of PCLA, PCLA/KH‐HA, PCLA/HA‐EGCG and PCLA/KH‐HA‐EGCG scaffolds were measured. 3D printed scaffold of 40 × 40 × 20 mm^3^ was put into a serum glass bottle, and add 15 ml of PBS solution (pH = 7.4). Scaffolds were printed 4 × 4 × 4 mm^3^ cubes, then the compression performance of the PCLA scaffold was tested by a dynamic and static material testing machine (Instron‐E3000, UK) under a 20 N detection probe based on GB/T 1041–2008. The compression speed of the samples is set to 1 mm/min, and three samples are tested to calculate Young's modulus of the PCLA scaffolds.

### 2.5 EGCG connection rate study

All the filtrate of initially prefabricated nano‐particles was collected together. EGCG loading percentage in nano‐particles was measured spectrophotometrically at 275 nm. The connection efficiency is calculated by the following equation:

(1) $$\;\text{EGCG loading content}\left(\%\mathrm{wt}/\mathrm{wt}\right)=\;\frac{\left(\text{mass of EGCG used}-\text{EGCG in sloution}\right)\times 100}{\text{mass of EGCG used}}.$$

### 2.6 Coating quantitative and stability study of scaffolds materials

Calcium ion content was used to highlight the coating content contained on the surface of the coated scaffold. The coated scaffolds (100 mg) were dissolved in dichloromethane, the precipitated material in the solution was collected by centrifugation, and then tested using a calcium assay kit (DICA‐048, Hayward, CA). To access the stability of the surface coating of the scaffold, PCLA/KH‐HA, PCLA/HA‐EGCG, and PCLA/KH‐HA‐EGCG scaffolds (100 mg) were immersed in 4 ml PBS solution, MEMα medium, or absolute ethanol for 7 days at 37°C. Subsequently, a quantitative analysis of the residual coating content on the surface of the coated scaffold was carried out according to the manufacturer's proposal.

### 2.7 Antibacterial ability test of scaffolds materials

To study the antimicrobial activity of PCLA, PCLA/KH‐HA, and PCLA/KH‐HA‐EGCG, the dilution coating plate experiment was performed. Briefly, a volume of 50 μl MRSA suspension was first evenly spread on the Mannitol Salt Agar Plate. Then, PCLA, PCLA/KH‐HA, and PCLA/KH‐HA‐EGCG were applied over the inoculated Mannitol Salt Agar Plate, respectively. Finally, the agar plates were incubated at 37°C for 24 h. On the other hand, standard plate counting assay was performed to assess antibacterial ability of these four materials. After being treated with PCLA, PCLA/KH‐HAZ, and PCLA/KH‐HA‐EGCG for 2 h at 37°C, MRSA samples were collected by centrifugation at 3600 rpm for 5 min. Then, samples were washed with PBS. Next, samples were serially diluted with sterile LB medium, and a volume of 50 μl samples were spread on agar plates. After incubation at 37°C for 48 h, colonies formed on the agar plates were photographed and counted.

### 2.8 Detection of ROS production

Briefly, MRSA cells were treated with PCLA, PCLA/KH‐HAZ, and PCLA/KH‐HA‐EGCG for 2 h in a shaker incubator. Next, MRSA cells were washed with PBS three times. Then the ROS level was detected by the addition of DCFH‐DA dye (Beyotime, China) at 10 μM. After 30 min incubation, MRSA cells were collected using a centrifuge and resuspended three times with PBS. Then, ROS production in MRSA cells was detected and imaged using a fluorescence microscope with the excitation wavelength at 488 nm.

### 2.9 Morphology observation of MRSA cells by SEM

After treatment with PCLA, PCLA/KH‐HAZ, and PCLA/KH‐HA‐EGCG scaffolds for 2 h in a shaker incubator, MRSA cells were collected using a centrifuge at 3600 rpm for 3 min at 4°C. Then, MRSA cells were fixed with 2.5% glutaraldehyde at 25°C for 2 h. Next, MRSA cells were washed with PBS three times. Then, samples were dehydrated by sequential treatment with 50%, 70%, 80%, 90%, 95% and 100% ethanol for 10 min each. After that, a drop of 5 μl dehydrated MRSA cells suspension was added on a silica wafer and air‐dried. Finally, the morphology of MRSA structure was obtained under an SEM (S‐4800, Hitachi, Japan) at an accelerating voltage of 10 kV.

### 2.10 Biomineralization performance test of scaffolds materials

For analysis of scaffold biomineralization performance, scaffolds were immersed in simulated body fluid (SBF) simulated body fluid for 14 days, and subsequently vacuumed drying before determining the deposited hydroxyapatite layer on the scaffold surfaces. The hydroxyapatite layer formed on the surface was observed by a scanning electron microscope.

### 2.11 Osteocyte proliferation and cell safety evaluation after scaffold stimulation

Mouse osteoblasts (MC3T3‐E1) were used to study the cell adhesion, and proliferation on PCLA, PCLA/KH‐HA, PCLA/HA‐EGCG, and PCLA/KH‐HA‐EGCG scaffold. MC3T3‐E1 cells were cultured in MEMα medium and 10% FBS with 1% penicillin and streptomycin at 37°C in an incubator with 5% CO_2_. Initially, scaffold samples were immersed in MEMα medium of 96‐well plates for 30 min to achieve complete surface infiltration. Then, MC3T3‐E1 cells (5 × 10^3^ cell/ml) were seeded on the scaffold samples and incubated for 24 h. Cell cytotoxicity, cell adhesion performance, proportion of live cells on the scaffolds were analysed by CCK‐8 regent and Live/Dead Cell kit, respectively. The cytotoxicity of different treatment groups was calculated by the following equation.

(2) $$\text{Cell viability}\;\left(\%\right)=\frac{\left(\mathrm{As}-\mathrm{Ab}\right)\times 100}{\mathrm{Ac}-\mathrm{Ab}},$$

where As: absorbance value of the scaffolds treatment group. Ac: Absorbance value of the control group without scaffolds. Ab: Absorbance value of the blank group without cells and scaffolds.

After the 1, 3, 5, and 10 days of co‐culture, cells attached to the scaffolds were stained with combination dye for 30 min according to the manufacturer's protocol, and then observed green of viable cells and red of dead cells fluorescence by fluorescence microscopy (Axio Observer 3 m, ZEISS, Germany). Additionally, the ratio of viable cells (3 days) was calculated by reading the absorbance values of 96‐well plates at 494 and 535 nm. On the other hand, A549 and HepG2 cells were seeded in a 6‐well plate and incubator for 12 h. Next, cells were treated with PCLA, PCLA/HA‐EGCG, PCLA/KH‐HA, and PCLA/KH‐HA‐EGCG for 24 h. Then, these samples were fixed with 3.7% paraformaldehyde and stained using 0.1% crystal violet, respectively. Next, samples were imaged under a microscope. Furthermore, we performed wound healing assay to evaluate the effect of scaffolds materials on cell motility. Briefly, A549 and HepG2 cells were seeded into 6‐well plate. When cell density of the culture dish reaches 90%, a 200 μl pipette tip was used to draw a straight line in the middle of the culture dish. Next, cells were washed with PBS twice and treated with PCLA, PCLA/HA‐EGCG, PCLA/KH‐HA and PCLA/KH‐HA‐EGCG for 24 h. Then, samples were fixed with 3.7% paraformaldehyde and stained using 0.1% crystal violet. Finally, the gaps in the scratched cell layer were imaged using a light microscope. The scratch gap width was measured to assess the effect of the scaffolds materials on cell movement.

### 2.12 Detection of alkaline phosphatase activity and osteogenic differentiation factor

Mouse MC3T3‐E1 cells were cultured in standard media in the presence of PCLA, PCLA/KH‐HA, PCLA/HA‐EGCG, and PCLA/KH‐HA‐EGCG scaffolds as mentioned above. After 3 and 7 days of culturing on the scaffolds samples, the cells were lysed with RIPA lysis buffer, centrifuged, and the supernatant was collected into a 96‐well plate. Before detecting alkaline phosphatase (ALP) activity in the supernatant at a wavelength of 405 nm (Beyotime, China), BCA protein kit was used for sample homogenization. Concentration of Osteocalcin (OCN), osteopontin (OPN), and Collagen type I (COL‐I) in the cell lysate supernatants from each experiment at 7 days were measured by ELISAs using OCN, OPN, and COL‐I ELISA kits (FANKEW, China) according to the manufacturer's protocol.

### 2.13 RT‐PCR

The pre‐sterilized scaffold samples were placed into a 6‐well plate with 2 ml/well of fresh MEMα medium to soak for 30 min. Then replace the medium used for infiltration with 2 ml of cell‐containing MEMα medium. After 48 h, the scaffold samples were transferred to a new 6‐well plate with fresh medium and cultured for 10 days. The total RNA was then extracted from the cells using Trizol reagent (Invitrogen, USA). Next, the RNA uses NovoScript®Plus All‐in‐one 1st Strand cDNA Synthesis SuperMix (gDNA Purge, Novoprotein) for reverse transcription, and then uses SYBR Green qPCR Master Mix (MedChemExpress) for real‐time PCR reaction through the real‐time PCR system (7500fast, ABI, China), according to the manufacturer's instructions. Each sample was tested in three parallels, and NADPH was selected as a reference. The primer sequences used in the experiment are described in Table S1.

### 2.14 Hemolysis assay

The potential toxicity of the PCLA scaffolds to mouse red blood cells was assessed using a standard protocol. An animal experiment was conducted in compliance with the Chinese Academy of Medical Sciences guidelines and was approved by the Institutional Animal Care and Ethics Committee (Approval No. SCXK2014‐0004). Briefly, a volume of 1 ml of blood collected from ICR mouse was put into the blood‐collecting vessel. Then, the blood cells were collected using a centrifuge at 1500 rpm for 3 min. Next, samples were washed with PBS and diluted to 10 ml. After that, 1 ml diluted red blood cell suspension solution was mixed with PCLA, PCLA/KH‐HA, PCLA/HA‐EGCG, and PCLA/KH‐HA‐EGCG scaffolds. These samples were then placed in a cell incubator at 37°C for 2 h. The positive control was added deionized water. Next, these scaffolds were removed from the samples before centrifugation. Finally, samples were photographed and the absorbance of supernatant at 570 nm was measured using a microplate reader to obtain the hemolysis percentage. The relative hemolysis percentage was calculated by the following equation:

(3) $$\text{Hemolysis}\;\left(\%\right)=\frac{\mathrm{Abs}\left(\text{sample}-\text{Blank}\right)\times 100}{\mathrm{Abs}\left(\text{Positive Control}-\text{Blank}\right)}.$$

### 2.15 Statistical analysis

All data were presented as mean ± SD (*n* = 4), and One‐way ANOVA was conducted to analyse statistically significant. *p* value <0.05,*, <0.01,** and <0.001,*** was considered significant. n.s, no significant. All data quantifications were done on high‐resolution images using Image Pro‐Plus6.0 software.

## 3 RESULTS AND DISCUSSION

### 3.1 Fabrication and characterization of KH‐HA‐EGCG nanoparticles

**FIGURE 2:**
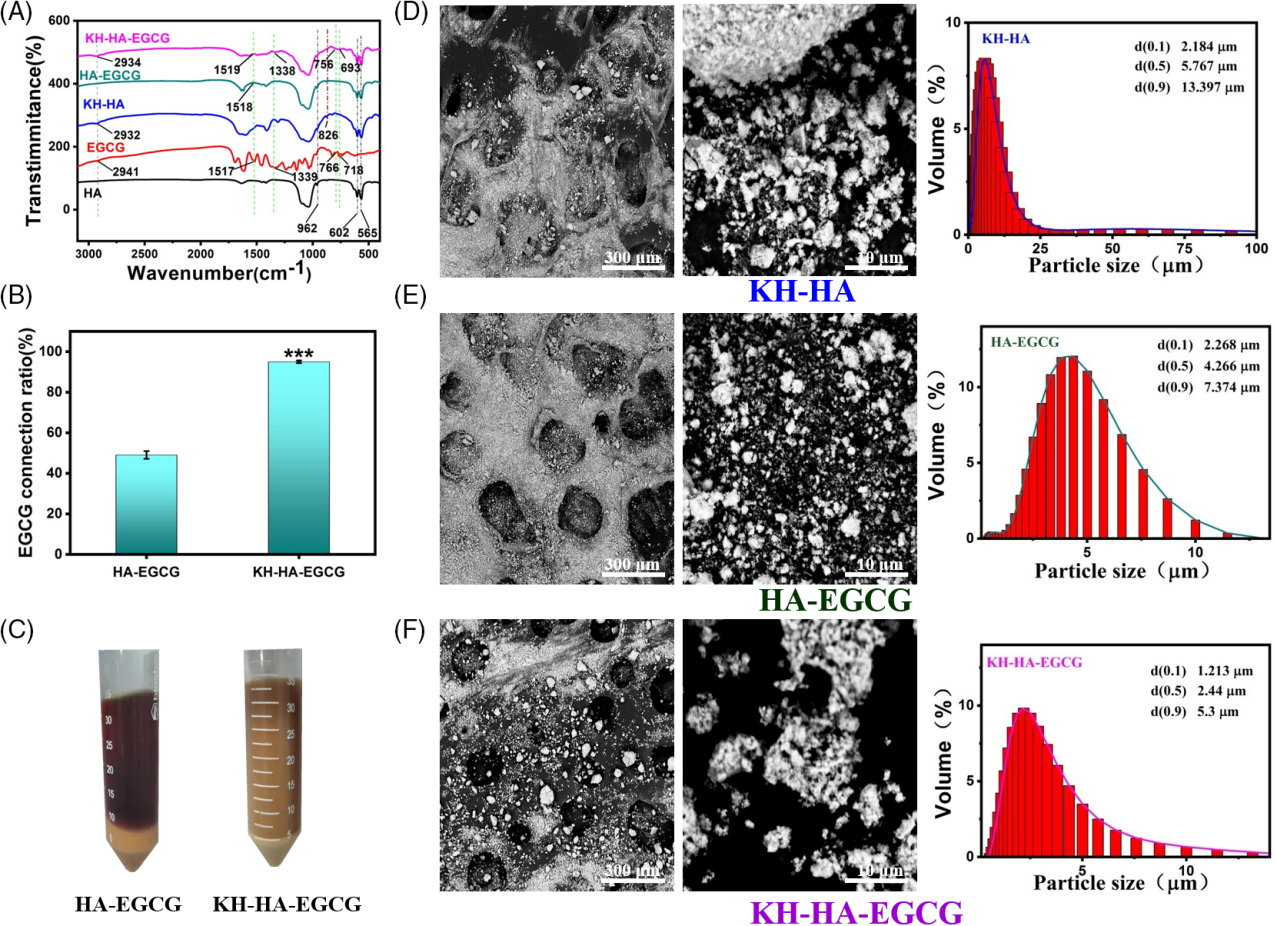
Characterization of different modified nano‐hydroxyapatite (HA). (A) Analysis of functional groups in different modified HA using FTIR. (B) EGCG connection rate in HA‐EGCG and KH‐HA‐EGCG nanoparticles. (C) Electronic photographs of HA‐EGCG and KH‐HA‐EGCG nanoparticles. (D) Left: Scanning electron micrograph of KH‐HA. Right: KH‐HA particle size analysis. (E) Left: Scanning electron micrograph of HA‐EGCG. Right: HA‐EGCG particle size analysis. (F) Left: Scanning electron micrograph of KH‐HA‐EGCG. Right: KH‐HA‐EGCG particle size analysis. Data are presented as the mean ± SD, ***p < 0.001. EGCG, epigallocatechin‐3‐gallate; FTIR, Fourier‐transform infrared

HA was partially condensed to produce a silicon‐oxygen covalent group on the HA backbone using a silane coupling agent (KH550). The functional groups in the prepared product were confirmed by infrared spectroscopy (Figure 2A). KH‐HA‐EGCG nanoparticles were subsequently fabricated by mixing KH‐HA and EGCG solutions. Amino group in the molecular chain of silane coupling agent reacted with EGCG to successfully modify EGCG on the surface of HA. As illustrated in Figure 2A, peaks at 2941, 2934, and 2932 cm^−1^ correspond to the asymmetric contraction vibration absorption of methyl CH_2_. As for spectroscopic peaks at 1517 and 1518 cm^−1^, they may be attributed to the C—H in‐plane bending vibration of the methylene group. In addition, 1338 and 1339 cm^−1^ correspond to hydroxyl O—H in‐plane bending vibration. 1519 cm^−1^ is C=C contraction vibration peak derived from the benzene ring of EGCG. The symmetrical contraction vibration peaks of HA‐specific phosphate groups at 602, and 565 cm^−1^ were observed in all samples. Most importantly, siloxy (Si—O) groups derived from trimethylsilane were observed at 826 cm^−1^. Moreover, as expected, EGCG content of the nanoparticles increased as KH550 was added, and KH‐HA‐EGCG exhibited good dispersibility (Figures 2B,C and S1–S3). After 8 times of centrifugation and purification, KH‐treated EGCG has been completely linked to HA. As shown in Figure S3, the supernatant is clear and transparent, while the supernatant of the group without KH550 treatment still contained unlinked EGCG. These results demonstrated that after a simple coupling method, a large amount of EGCG was successfully firmly attached to the surface of the HA nanoparticles.

Microscopic surface structure characteristics of modified HA were shown in Figure 2D–F. Compared with HA‐EGCG and KH‐HA nanoparticles, SEM image of modified HA nanoparticles show that KH‐HA‐EGCG had better dispersibility (Figure 2F). Additionally, particle sizes of KH‐HA, HA‐EGCG, and KH‐HA‐EGCG fabricated were also investigated. The particle sizes decreased as the EGCG content increased (Figure 2D–F). This may be attributed to the HA nanoparticle being firstly treated with the silane coupling agent to enhance their dispersibility, and then the amino group inside the silane coupling agent was bridging the EGCG to the HA surface. These results collectively proved that EGCG was successfully modified on the surface of HA, and KH‐HA‐EGCG had a higher EGCG intensity.

### 3.2 Fabrication and characterization of 3D coated scaffold

**FIGURE 3:**
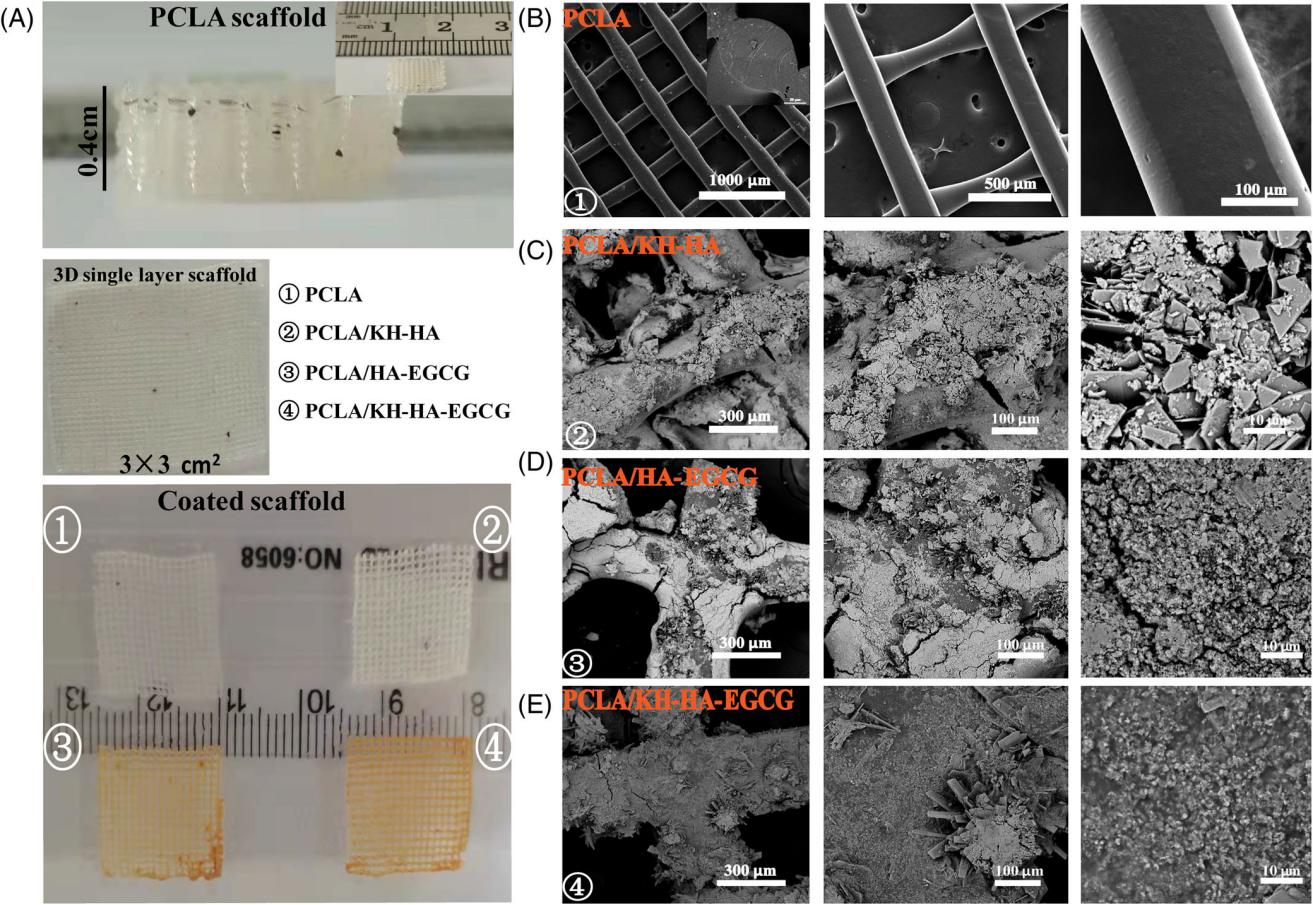
Characterization of different coated scaffolds. (A) Top: Representative photograph of 3D printed PCLA scaffold (the illustration above is the width of the scaffold). Middle: 3D printed single‐layer PCLA scaffold film. Bottom: Different coated scaffolds. (B) Representative SEM images of pure PCLA scaffold (the illustration shows the cross‐section of the PCLA scaffold). (C) Representative SEM images of PCLA/KH‐HA scaffold. (D) Representative SEM images of PCLA/HA‐EGCG scaffold. E, Representative SEM images of PCLA/KH‐HA‐EGCG scaffold. EGCG, epigallocatechin‐3‐gallate; PCLA, polymerization of caprolactone and lactide; SEM, scanning electron microscopy

The open pores in the scaffold directly affect the transport of nutrients and metabolites for bone tissue growth, as well as the formation of new blood vessels.^30^, ^31^, ^32^ Our previous research reported that the prepared PTMC/KHA/VH microsphere scaffold had 42% porosity and 100 μm pore size using microsphere sintering technology.^28^ This PTMC/KHA/VH microsphere scaffold presented bone repair performance with good biocompatibility in vitro and in vivo. However, the PTMC/KHA/VH scaffold had poor repeatability due to the microspheres' wide particle size distribution range of the microspheres. The application of 3D printing technology can accurately customize any geometrical bone repair for the patient's injury site, and can also effectively control and optimize the porous structure of the scaffold to optimize bone healing.^33^ In this study, we fabricated the scaffolds using a 3D printer with a nozzle diameter of 200 μm. The PCLA scaffold was firstly manufactured in a prefabricated mesh model and used 0/90° or 0/45° mode to continuously extrude polymer filaments under a pressure of 200 kpa to ensure the 3D porous space structure of the printed scaffold. This process was used as a single‐layer printing of the scaffold (Video S1). After many times layer‐by‐layer printing and assembly, a 3D scaffold with a height of millimetres or centimetres can be quickly printed.^34^ During the coating process, three scaffolds were manufactured for further analysis. They were the pure PCLA/KH‐HA scaffolds, PCLA/HA‐EGCG scaffolds, and PCLA/KH‐HA‐EGCG scaffolds. In addition, the uncoated PCLA scaffold served as a blank control. The appearance of the PCLA scaffold and the coated scaffold was displayed in Figure 3A (top and bottom). As shown in Figure 3B, cross‐sectional and surface morphology images showed the microstructure features of the PCLA scaffold (left). Compared with the colourless and transparent PCLA group, the PCLA/HA‐EGCG and PCLA/KH‐HA‐EGCG scaffolds showed a clear brown rough surface (Figure 3A). This is due to the self‐assembly of EGCG in alkaline solution into brown nanostructures and rivets on the surface of the PCLA scaffold with the aid of KH550. The SEM images showed that the PCLA scaffold was smooth and flat on the surface and inside, and the inside of the scaffold was highly interconnected (Figure 3B). On the contrary, it is obvious that the surface of the coated scaffold is very rough, and the presence of HA could be observed. Interstingly, PCLA/KH‐HA‐EGCG scaffold exhibits a more uniformly dispersed microstructure (Figure 3C–E). This is because the smaller the particle size of KH‐HAZ‐EGCG, the better its dispersion in the solution at the same concentration.

**FIGURE 4:**
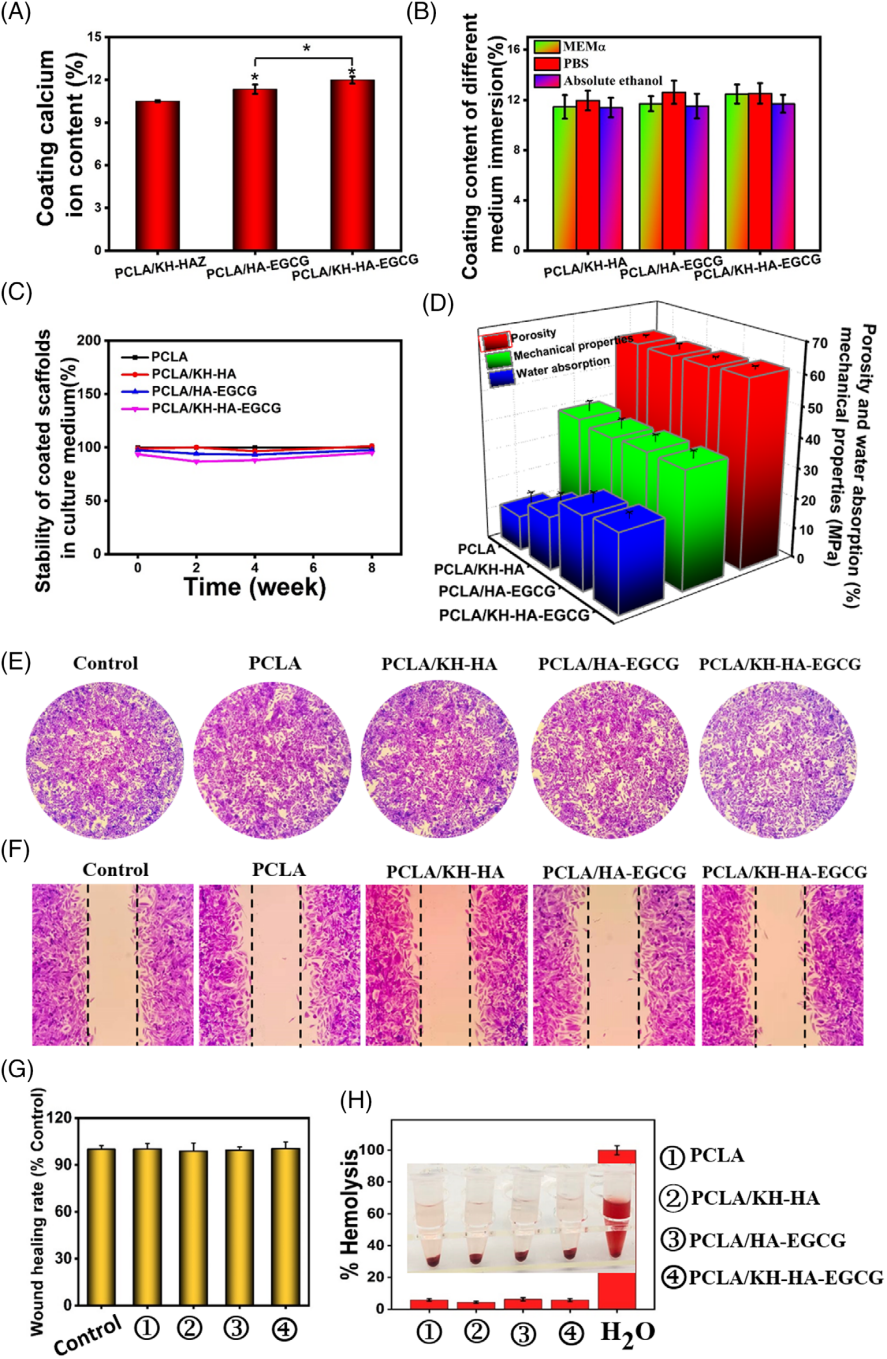
Characterization of the stability and biosafety of the coated scaffolds. (A) Quantitative detection of the coating content on the surface of the scaffold. (B) Testing the stability of coatings in different media (MEMα, phosphate buffered saline, and absolute ethanol). (C) Stability testing of coated scaffolds in culture medium for 8 weeks. (D) Testing the porosity, water absorption, and mechanical properties of scaffolds. (E) Typical representative photographs of HepG2 cells counts after treated with scaffolds. (F) Typical representative photographs of migration of HepG2 cells after treatment with Scaffolds. (G) Quantitative statistics of the scratch gaps of HepG2 cells corresponding to F. (H) Hemolysis analysis. H_2_O was used as a positive control. The inset represents a visual photograph after adding different scaffolds and incubating for 2 h. Data are presented as the mean ± SD, *p < 0.05.

Based on the different appearances of the scaffold coating, we further quantified the coating content of the 3D printed PCLA scaffold surface. The 3D scaffold sample matched the coating very well during the coating process. As depicted in Figure 4A, calcium ion content was quantified to evaluate the coating efficiency of modified PCLA scaffolds. PCLA/HA‐EGCG and PCLA/KH‐HA‐EGCG groups presented higher concentrations of calcium ions than PCLA/KH‐HA (Figure 4A). This was because the particle size of HA‐EGCG and KH‐HA‐EGCG were smaller (Figure 2D–F), and the amount of HA adhered to the PCLA scaffolds was higher at the same concentration. EGCG‐modified HA coating designed in this study could stably exist on the surface of the scaffold to achieve long‐term effects. As excepted, the quantitative results of the coating showed that the coating could stably exist on the surface of the scaffold, whether in PBS buffer, MEMα medium, or absolute ethanol and could maintain stability for a long time (Figures 4B,C, S4, and S5). The Young's modulus of PCLA scaffold was 39.3 ± 3.2 MPa, which has similar mechanical properties to cartilage (20‐100 MPa; Figure 4D).^35^ Notably, the coating method designed in this study could be useful for any 3D printed scaffold with different applications. Furthermore, the 3D scaffold samples' porosity was measured using absolute ethanol replacement technology. The porosities of PCLA, PCLA/KH‐HA, PCLA/HA‐EGCG and PCLA/KH‐HA‐EGCG scaffolds were 60.7 ± 0.6%, 60.4 ± 1.7%, 60.8 ± 0.6%, and 61.6 ± 0.9%, respectively (Figure 4D). There was no significant difference in pore connectivity and porosity between the scaffolds with different coatings. The water uptake of scaffolds fabricated with various coating was also investigated. The application of the coating for the surface of the scaffold fabricate had enabled increased scaffold's water absorption performance to optimize buffer infiltration and facilitate cell adhesion. As shown in Figure 4D, the water absorption of the 3D scaffold increased when the coating was KH‐HA‐EGCG nanoparticles. This is because the rough structure makes the PCLA scaffold surface have a larger specific surface area, making it difficult to remove excess water. Moreover, HA has a strong ability to absorb water and release water and absorb moisture in the air.^36^ This increase in water absorption is different from our previous studies on the water absorption of the bone repair scaffold with the incorporation of modified inorganic particles.^28^

On the other hand, we evaluated the cytotoxicity of these coated scaffolds. As depicted in Figure 4E, after treatment with different coating PCLA scaffolds, the count of HepG2 cells did not decrease, indicating that these scaffolds had good biosafety for HepG2 cells. Cell motility is an important indicator for evaluating cell viability.^37^, ^38^ Wound‐healing studies showed that the motility of HepG2 cells was not affected by different PCLA scaffolds treatment, further proving that these scaffolds were not toxic to cells (Figure 4F,G). As expected, coated scaffolds did not exhibit cytotoxicity to A549 cells (Figure S6). Furthermore, we used a hemolysis experiment to evaluate the toxicity of scaffolds materials on the rupture and lysis of red blood cells in ICR mouse. PCLA/KH‐HA‐EGCG did not cause obvious hemolysis, indicating their excellent blood biocompatibility (Figure 4H).

### 3.3 PCLA/KH‐HA‐EGCG scaffold inhibits bacteria in vitro

**FIGURE. 5:**
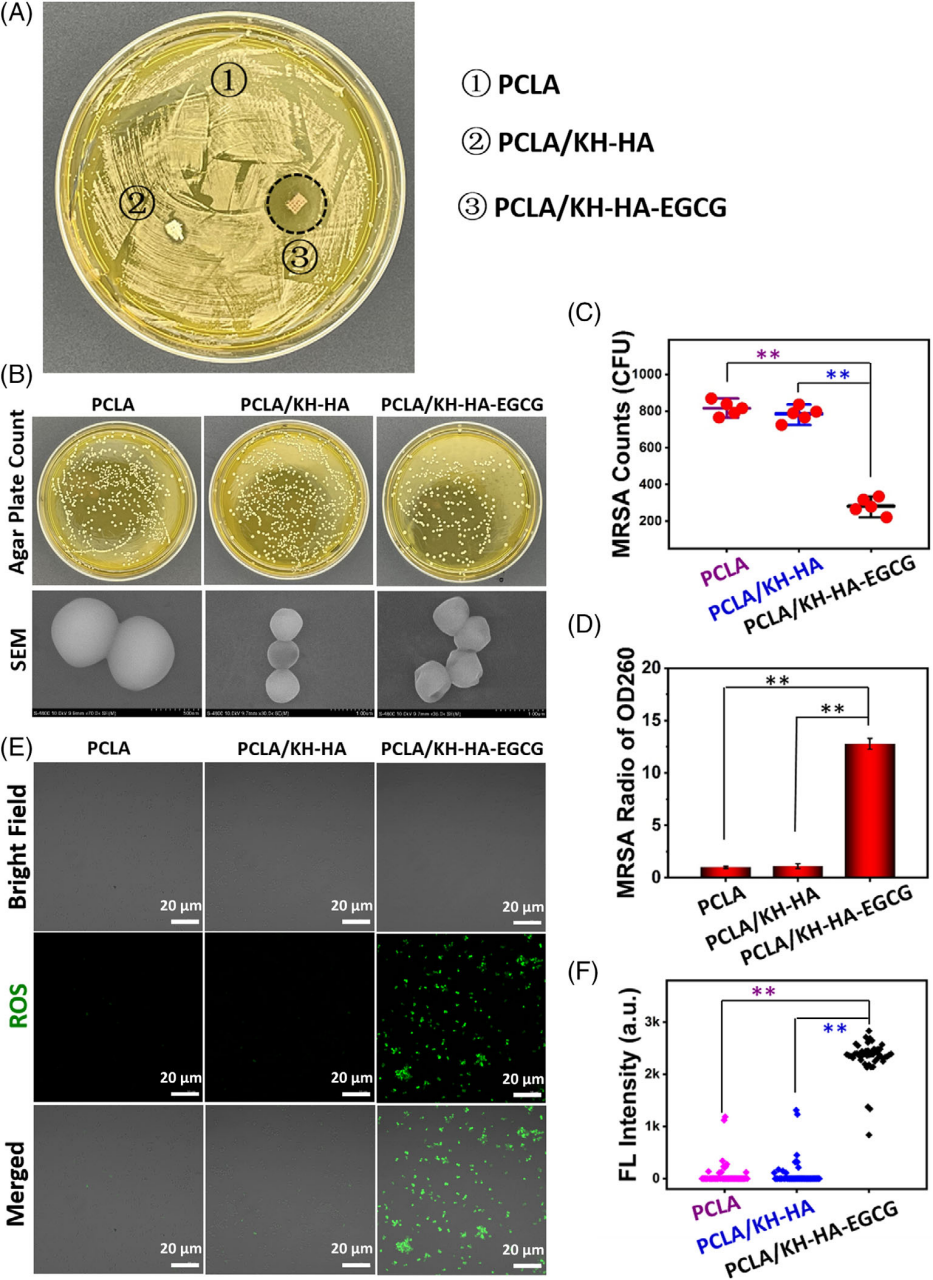
Antimicrobial activity of the coated scaffolds. (A) The zone of inhibition (ZOI) study of different PCLA scaffolds for MRSA. (B) The intuitive agar plate counts and SEM images of structural integrity of the treated MRSA in different groups. (C) Agar plate count statistics of MRSA corresponds to B. (D) The content of nucleic acids in MRSA culture medium quantified after treatment with different PCLA scaffolds. (E) Fluorescence staining shows the burst of ROS in MRSA after treatment with different PCLA scaffolds. (F) Fluorescence intensity analysis corresponds to E. Each point represents the fluorescence intensity of an individual MRSA cell. Data are presented as the mean ± SD, **p < 0.01. MRSA, methicillin‐resistant *Staphylococcus aureus*; PCLA, polymerization of caprolactone and lactide; ROS, reactive oxygen species; SEM, scanning electron microscopy

*Staphylococcus aureus* (SA) is one of the main causes of osteomyelitis. Moreover, multiple drug‐resistant bacteria MRSA were often detected clinically, posing a threat to human health.^39^, ^40^, ^41^ In this study, the zone of inhibition (ZOI) study was performed to assess the anti‐MRSA activity of prepared materials.^42^ As depicted in Figure 5A, PCLA, PCLA/KH‐HA and PCLA/KH‐HA‐EGCG were put on an agar plate that had been inoculated with MRSA. Results demonstrated that PCLA/KH‐HA‐EGCG displayed a significant zone of inhibition against MRSA, indicating the cumulative diffusion of it to the surroundings and killed MRSA in situ. PCLA/KH‐HA‐EGCG displayed a zone of inhibition with a diameter of 18 mm to MRSA, while PCLA and PCLA/KH‐HA did not show obvious antibacterial ability against MRSA (Figure 5A). Furthermore, after MRSA was treated with these PCLA scaffold materials, it was spread on agar plates to form colony‐forming units (CFU) and counts. As shown in Figure 5B,C, the CFU counts on the MRSA plate treated with PCLA/KH‐HA‐EGCG were the least, indicating the excellent antibacterial ability of PCLA/KH‐HA‐EGCG.

To investigate the antibacterial mechanism of the coated scaffolds, the morphology and structural integrity of the treated MRSA in different groups were observed by SEM. As depicted in Figure 5B, the wall and membrane structure of MRSA treated with PCLA/KH‐HA‐EGCG had been seriously destroyed. When the structure of bacteria is damaged, its intracellular components will flow out of the cell, such as nucleic acid and proteins. As shown in Figure 5D, the greatest amount of nucleic acid was detected in the culture medium of MRSA treated with PCLA/KH‐HA‐EGCG, further demonstrating the severe destruction of MRSA structure. Furthermore, we found that PCLA/KH‐HA‐EGCG promote the outbreak of reactive oxygen species (ROS) in MRSA cells (Figure 5E,F). ROS in MRSA is an important signal molecule. When the production of excessive ROS exceeds the scavenging ability of MRSA itself, it will cause fatal damage to the bacteria. Excessive ROS will attack important macromolecules in bacterial cell (e.g., nucleic acid, proteins and lipid), and eventually cause cell death.^43^, ^44^ As depicted in Figure 5E,F, the level of ROS in MRSA was significantly increased after PCLA/KH‐HA‐EGCG treatment, while no obvious ROS signal was observed in MRSA treatment with PCLA/KH‐HA without EGCG modified. These results collectively demonstrated that the EGCG‐modified scaffolds had good stability and biosafety, and could effectively induce endogenous ROS burst, destroy MRSA wall structure, and ultimately lead to bacterial death (Figures 4 and 5). These properties provide advantages for its clinical application in promoting bone regeneration and combating postoperative infection in situ.

### 3.4 Osteocyte proliferation and biomineralization of modified scaffolds

**FIGURE 6:**
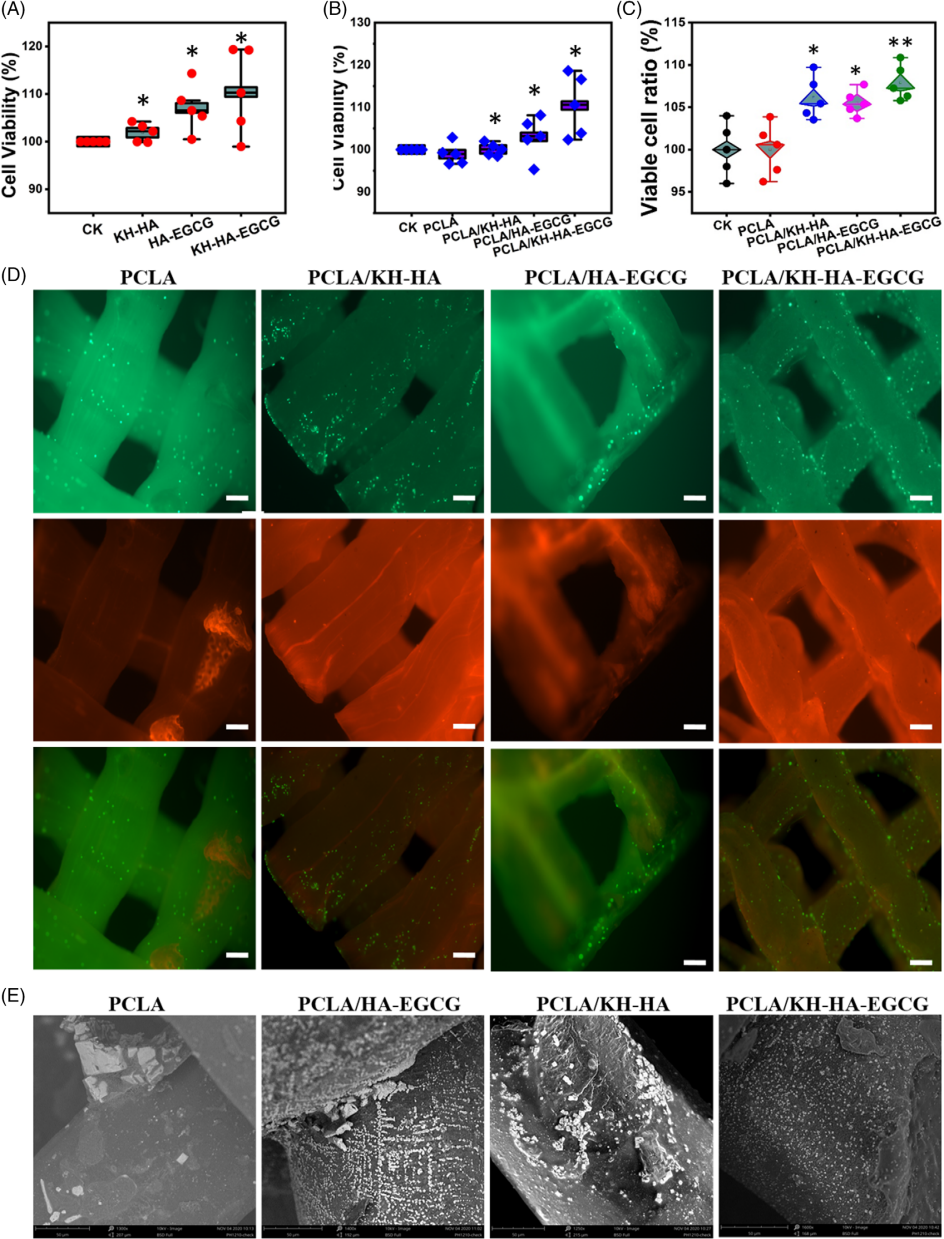
Osteocyte proliferation and biomineralization of the coated scaffolds. (A) Proliferation of MC3T3‐E1 osteoblast cells treated with different HA nanoparticles. (B) Proliferation of MC3T3‐E1 osteoblast cells treated with different PCLA scaffolds. (C) The ratio of the number of viable cells after 3 days in the fluorescent staining of cells. (D) Fluorescence microscopy images demonstrating MC3T3‐E1 cell proliferation with different scaffold samples (10 days). Scale bar (50 μm). (E) Surface hydroxyapatite deposition caused by different scaffold groups measured by biomineralization. Data are presented as the mean ± SD, *p < 0.05, **p < 0.01. HA, hydroxyapatite; PCLA, polymerization of caprolactone and lactide

The ideal bone scaffold material is capable of promoting the growth of osteoblast cells. The proliferation of MC3T3‐E1 osteoblast cells in the presence of inorganic particles was detected in vitro. Compared with the blank control group, the number of MC3T3‐E1 cells stimulated by KH‐HA, HA‐EGCG, and KH‐HA‐EGCG nanoparticles was increased at the same coating content concentration (Figure 6A). This is mainly because the Ca^2+^ and PO_4_^3−^ ions released by HA are beneficial to stimulate the proliferation of bone cells. Moreover, HA‐modified PCLA scaffolds also had the ability to promote MC3T3‐E1 osteoblast cells proliferation. As shown in Figure 6B, MC3T3‐E1 cells showed significant proliferation after PCLA/KH‐HA, PCLA/HA‐EGCG, and PCLA/KH‐HA‐EGCG scaffolds treatment. We also performed a live cell ratio test to investigate the detailed cell survival rate on the scaffolds, which showed PCLA/KH‐HA‐EGCG scaffold presented an excellent proliferation state (Figure 6C). To further intuitively reveal the effect of scaffolds on MC3T3‐E1 cell proliferation activity, we cultured mouse MCT‐E1 cells with different coated scaffolds and imaged them with a fluorescence microscope. After culturing for 1, 3, 5 (Figure S7) and 10 days (Figure 6D), live/dead staining showed that the cells seeded on the coated scaffold had more proliferation (green), and few dead cells (red) were observed. In this study, HA is the main component of human bone tissue, and it is also the main bio‐inorganic particle used in the current research on promoting bone regeneration.^45^ When it is compounded on the surface of PCLA scaffold as a coating, calcium and phosphorus will be freed from the surface of the coated scaffolds. The calcium and phosphorus will be absorbed by adherent cells, stimulating cell differentiation and even new tissue formation. Together, we demonstrate that after adding EGCG‐modified HA coating on the surface of the scaffold, the 3D printed PCLA scaffold could effectively promote cell adhesion and proliferation.

Furthermore, we analysed the biomineralization efficiency of the scaffold by observing the formation of the hydroxyapatite layer on the surface of the scaffold in a SBF environment, which was an essential feature of an ideal bone repair scaffold.^46^ The biomineralization ability of the scaffold can accelerate the initial repair process, where the hematoma formed at the bone defect site is usually used for cell migration and proliferation. The biomineralization results showed that after 14 days of culture, the number of HA cubes in PCLA/KH‐HA‐EGCG group was more than other groups (Figure 6E). The accumulation of the HA layer in the medium is closely related to the dispersibility of the HA in the scaffold coating. The more uniform coating leads to the HA layer being easier to enrich on the scaffold surface which was consistent with the results of the SEM image of the coated scaffold.

### 3.5 PCLA/KH‐HA‐EGCG scaffold promotes osteogenic differentiation of mouse MC3T3‐E1 in vitro

**FIGURE 7:**
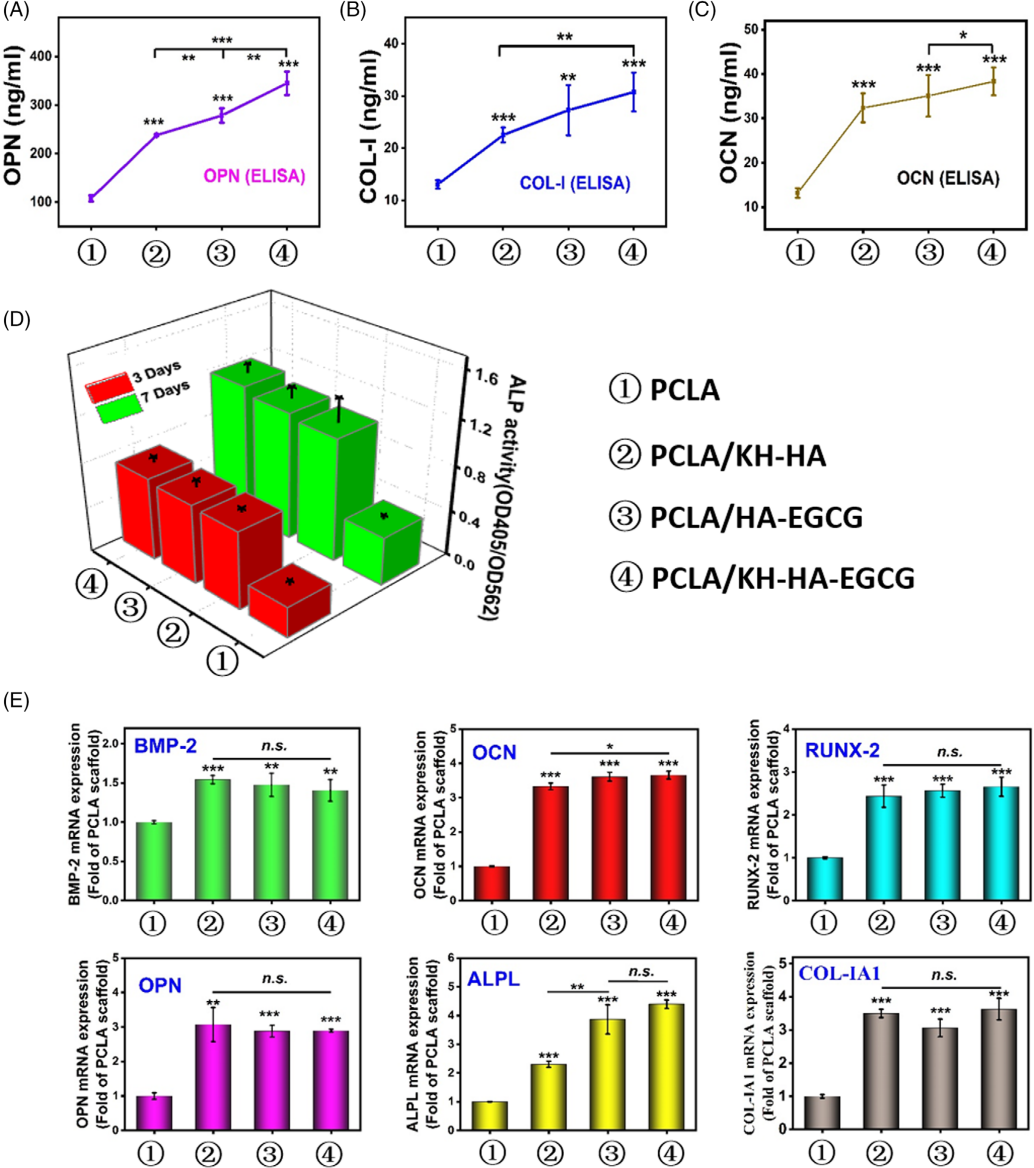
The effect of scaffold on mouse MC3T3‐E1 osteogenic related genes and protein expression. (A) The effect of different scaffold groups on osteopontin (OPN) protein expression. (B) The effect of different scaffold groups on type I collagen (COL‐I) protein expression. (C) The effect of different scaffold groups on osteocalcin (OCN) protein expression. (D) The effect of the scaffold on the ALP activity (OD 405 nm) of co‐cultured cells on the 3rd and 7th day. ALP activity is homogenized by total cell protein (OD 562 nm). (E) Statistic was showing the effects of different scaffold groups on the mRNA expression of osteogenic differentiation‐related genes, including bone morphogenetic protein 2 (BMP‐2), runt‐related transcription factor 2 (Runx‐2), osteocalcin (OCN), and osteopontin (OPN), type I collagen (COL‐I), Alkaline phosphatase (ALPL) after 10 days of culture. Data are presented as the mean ± SD, *p < 0.05, **p < 0.01, ***p < 0.001.

Osteocalcin (OCN), Osteopontin (OPN), and Collagen type I (COL‐I) proteins are essential in the process of bone formation.^47^ To clarify the effect of the PCLA/KH‐HA‐EGCG scaffold on the osteogenic differentiation‐related proteins in MC3T3‐E1 cells, OCN, OPN, and COL‐I peoreins were analysed. Cells cultured in PCLA scaffold showed a low gene expression level, which served as a negative control. Results displayed that the above three osteogenic differentiation‐related genes were all significantly up‐regulated in the scaffold coating group than that in PCLA group at 7 days (Figure 7A–C). When cells are co‐cultured with the coated scaffold, the protein production tends to increase with time prolonged (Figure S8). Similarly, compared with other coated scaffolds, the presence of PCLA/KH‐HA‐EGCG scaffold resulted in a higher expression of bone‐related proteins at day 7 (Figure 7A–C). To verify the addition of HA coating on the surface of the PCLA scaffold possess more effectively promote cell proliferation and differentiation, we conducted intracellular alkaline phosphatase (ALP) activity measurements. The ALP activity of mouse MC3T3‐E1 was examined on days 3 and 7 (Figure 7D). According to the normalized quantitative data analysis, cells seeded in the PCLA group showed significantly lower ALP activity after the cell culture period. The ALP activity of all scaffold groups was the same on the first day and gradually increased with the prolonged culture time. Modified scaffold groups presented higher ALP activity on the 3th and 7th days compared to the uncoated scaffold (Figure 7D). These results indicate that PCLA/KH‐HA‐EGCG scaffold‐treated MC3T3‐E1 cells had strong metabolic activity, further demonstrating its ability to promote osteoblast cells growth.

Consistently, mRNA expression results demonstrated that all osteogenic differentiation‐related genes, including OCN, OPN, Runt‐related Transcription Factor 2 (Runx2), Bone Morphogenetic Protein (BMP‐2), Collagen type I (COL‐IA1), and Alkaline phosphatase (ALPL) were significantly up‐regulated in PCLA/KH‐HA, PCLA/HA‐EGCG, and PCLA/KH‐HA‐EGCG group than that in PCLA group for 10 days (Figure 7E). These results indicated that adding EGCG‐modified HA to the 3D printed scaffold enhanced its osteoinductive ability compared to the PCLA without modification. Furthermore, PCLA/KH‐HA‐EGCG scaffold significantly up‐regulated the expression of genes and proteins related to osteogenic differentiation of the MC3T3‐E1 cells. Hence, PCLA/KH‐HA‐EGCG scaffold could potentially mimic bone systems to repair bone damage in regenerative medicine, as it inhibited bacteria colonization and stimulated cell differentiation in situ.

## 4 CONCLUSIONS

We demonstrated that manufacturing a 3D structural scaffold using an extrusion‐based printing method and imparting an EGCG‐modified HA coating on its surface could effectively enhance the scaffold's water absorption, osteogenic induction and antibacterial properties in situ. In this study, simple HA mixture solutions were used to disperse modified nanoparticles and prepare the coating without any other cross‐linking of the excipient. Interestingly, PCLA/KH‐HA‐EGCG scaffold exhibited a more uniform surface, excellent biomineralization properties and effectively promoted bone cell adhesion and proliferation. Moreover, PCLA/KH‐HA‐EGCG scaffold exhibited promising in situ antibacterial effects by trigging intracellular ROS burst and disrupting MRSA wall structure. Notably, the scaffold had excellent stability under different physiological environments in vitro and had good biocompatibility. Hence, this simple and universal EGCG‐modified HA‐coated PCLA scaffold proposal holds great promise in 3D printing‐related tissue engineering and bone infection repair research.

## AUTHOR CONTRIBUTIONS

X. Zhang., and J. He. performed the study and wrote the draft; L. Qiao., and Z. Wang. synthesized and characterized PCLA materials. Q. Zheng., C. Xiong. and H. Yang. performed the material characterization and cell analysis. K. Li., C. Lu., and S. Li. participated in data analysis; H. Chen. and X. Hu. proposed the research idea and revised the manuscript. All authors have read and agreed to the published version of the manuscript.

## CONFLICT OF INTEREST

The authors declare no conflict of interest.

## Supporting information


**Appendix S1** Supporting information.

Video S1

## Data Availability

The data that supported the findings of this study are available on request from the corresponding authors.
