# Supplementary material for: 3D printed PCLA scaffold with nano‐hydroxyapatite coating doped green tea EGCG promotes bone growth and inhibits multidrug‐resistant bacteria colonization
Source: Cell Prolif. 2022 Jul 5;55(10):e13289. doi: 10.1111/cpr.13289 (PMC9528762; doi:10.1111/cpr.13289)
Supplement: Supplementary file 1 — Appendix S1 Supporting information. [file CPR-55-e13289-s002.docx]

**3D printed PCLA scaffold with nano-hydroxyapatite coating doped with green tea polyphenols promotes bone growth and inhibits multdrug-resistant bacteria colonization**

Xiangchun Zhang^1†^, Jian He^3†^, Liang Qiao^4^, Ziqi Wang^1^, Qinqin Zheng^1^, Chengdong Xiong^5^, Hui Yang^6^, Kainan Li^2^, Chengyin Lu^1^, Sanqiang Li^3^, Hongping Chen^1^*, Xulin Hu^2^*

^1^ Tea Research Institute, Chinese Academy of Agricultural Sciences, Hangzhou 310008, China.

^2^ Clinical Medical College & Affiliated Hospital of Chengdu University, Chengdu University， Chengdu 610081, China.

^3^ College of Medical, Henan University of Science and Technology, Luoyang 471023, China.

^4^ The First Affiliated Hospital, College of Clinical Medicine of Henan University of Science and Technology, Luoyang, 471003, P.R. China.

^5^ Chengdu Institute of Organic Chemistry, Chinese Academy of Sciences, Chengdu, Sichuan 610041, China

^6^ State Key Laboratory of Oral Diseases & National Clinical Research Center for Oral Diseases & West China Hospital of Stomatology, Sichuan University, Chengdu 610041, China

*To whom correspondence should be addressed,

E-mail: [huxulin1993@163.com](mailto:huxulin1993@163.com), [thean27@tricaas.com](mailto:thean27@tricaas.com)


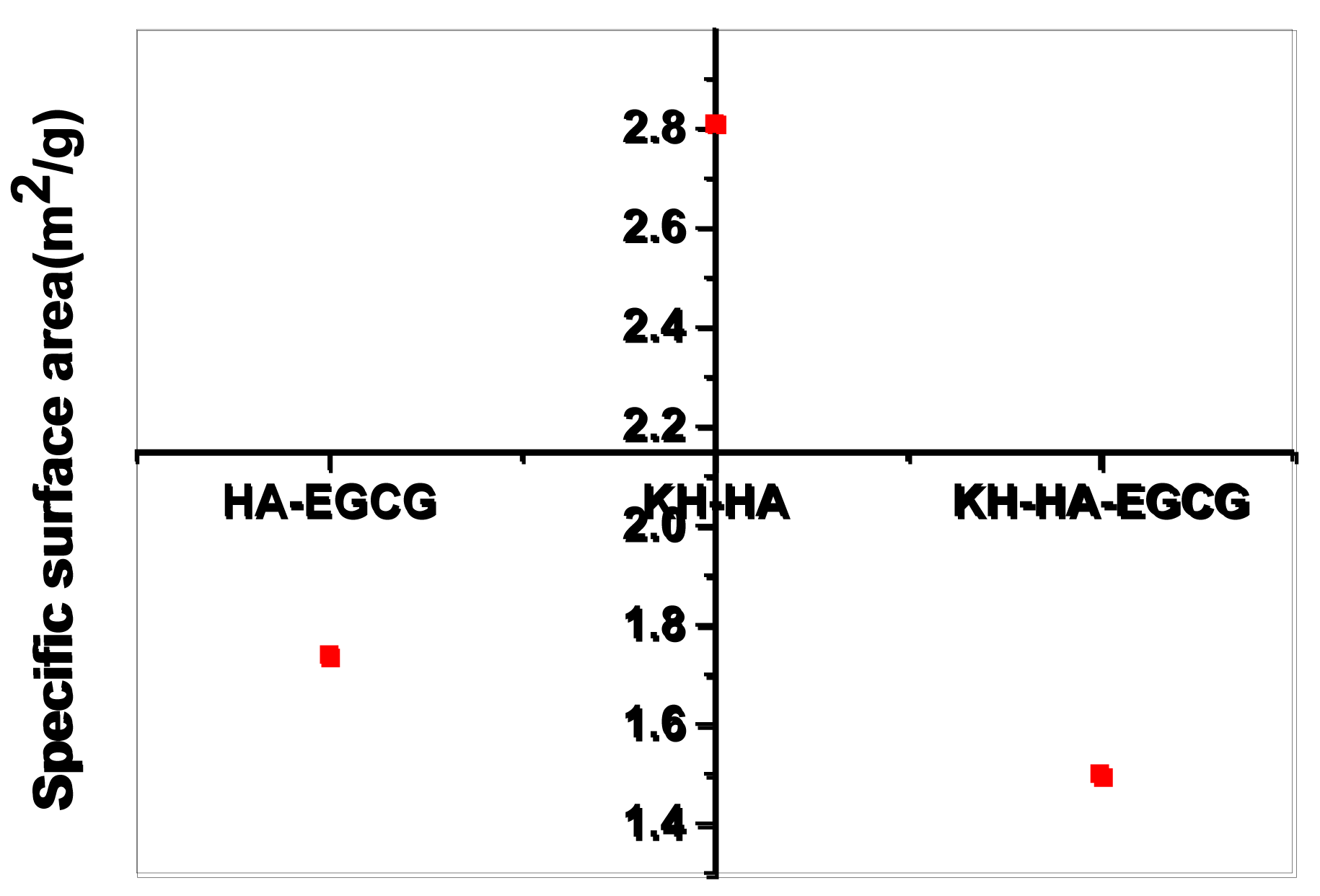


**Figure S1.** Statistics on the change of specific surface area before and after hydroxyapatite modification. The smaller the particle size, the smaller the specific surface area.

**
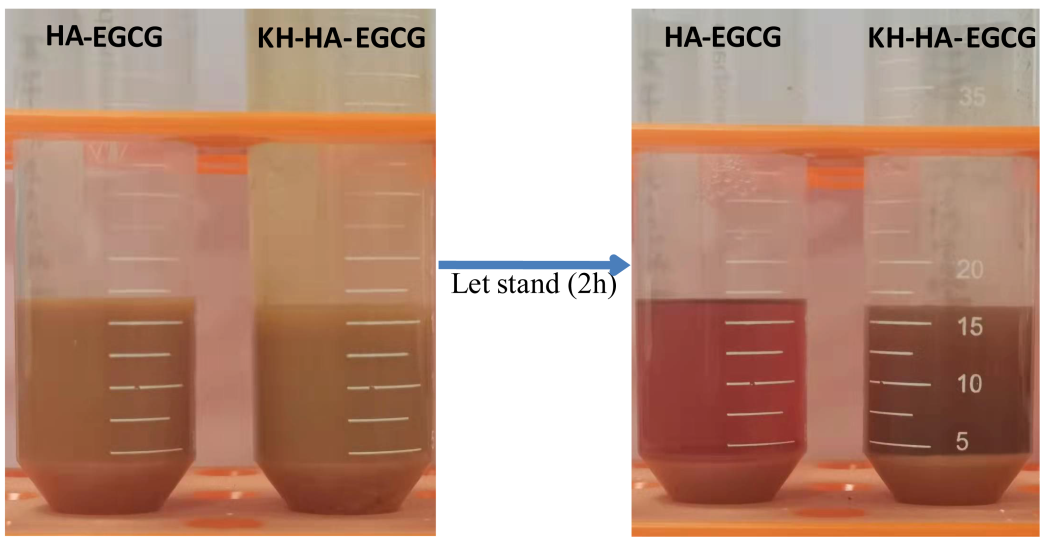
**

**Figure S2.** Comparison of preliminary samples of hydroxyapatite modified with and without coupling agent. The presence of coupling agent increases the amount of EGCG attached.

**
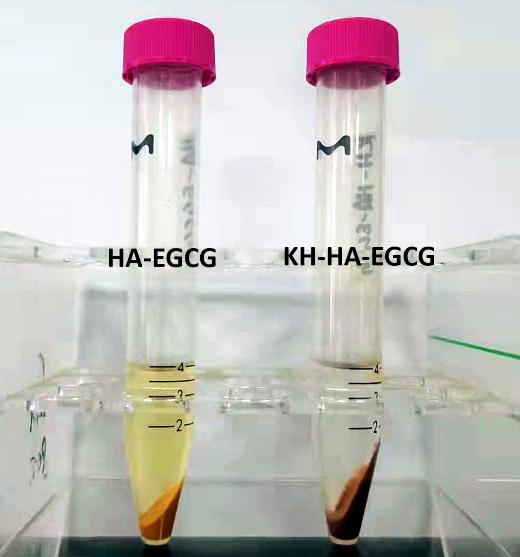
**

**Figure S3.** The color of the centrifugal fluid changes after purification with and without coupling agent modified hydroxyapatite. The centrifuge solution without coupling agent is obviously yellow.

**
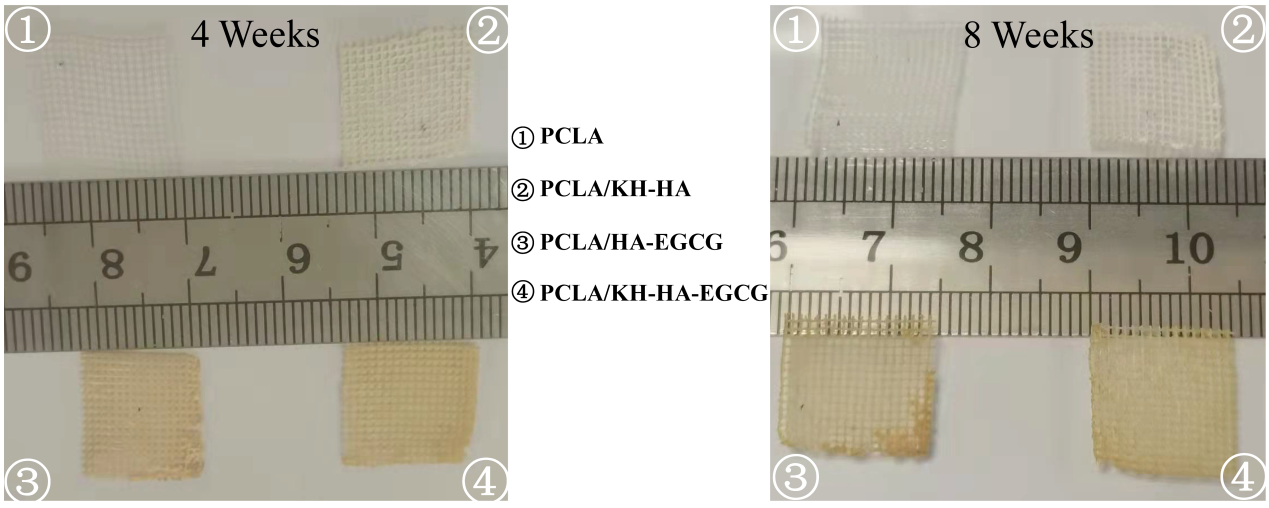
**

**Figure S4.** The stability of the surface coating of the coated scaffold was observed after being soaked in alcohol for 4 weeks and 8 weeks (4°C).


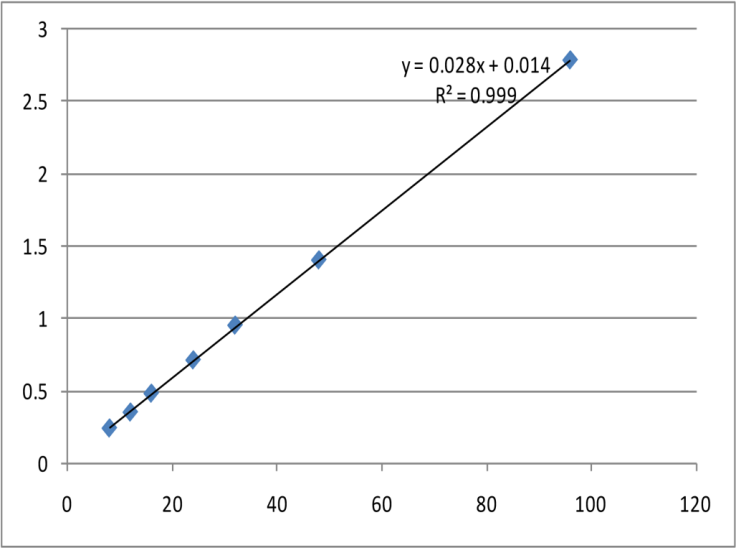


**Figure S5.** EGCG standard curve in the EGCG connection rate detection experiment.

**
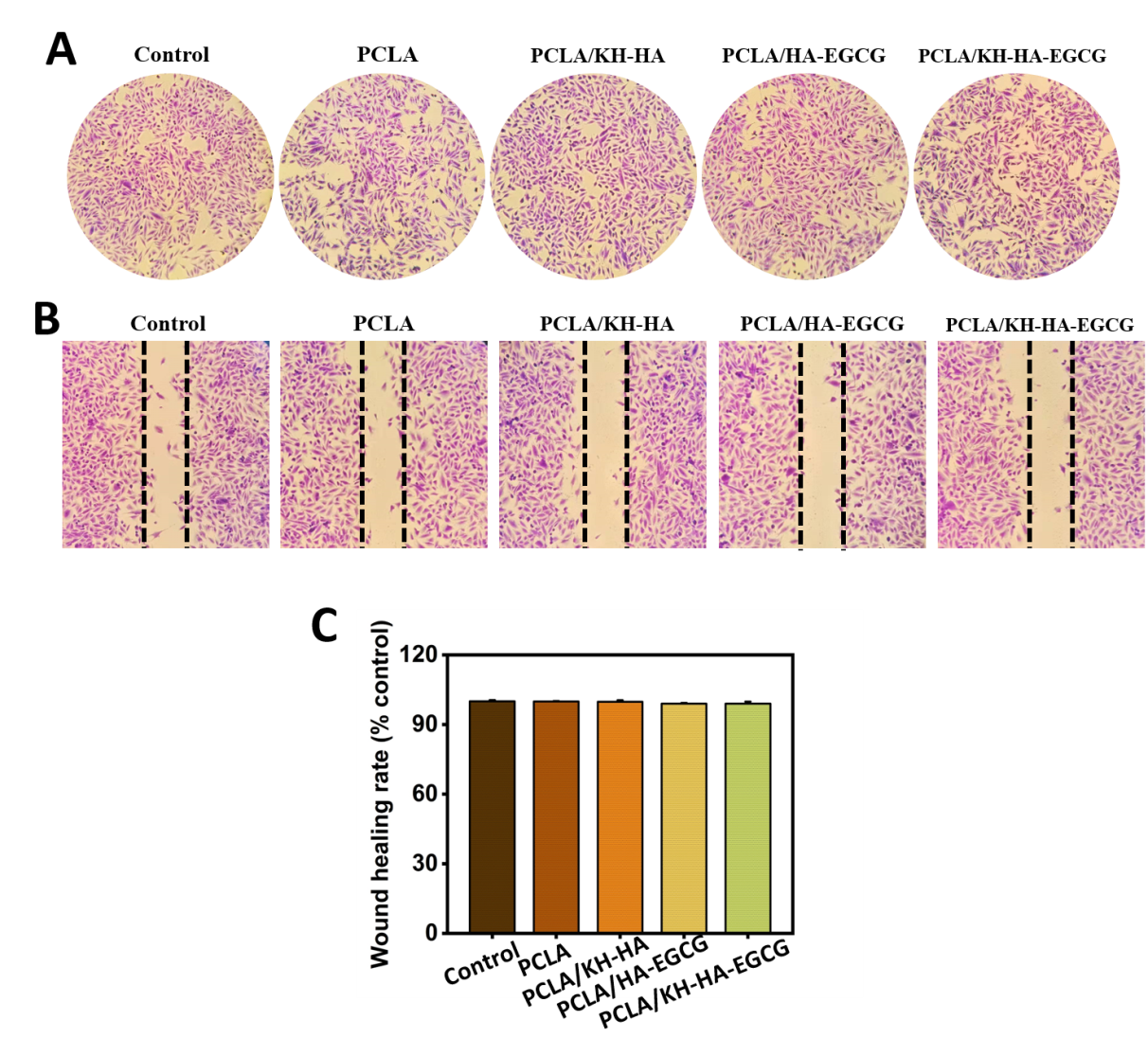
**

**Figure S6.** A, Typical representative photographs of A549 cells counts after treated with scaffolds. B, Typical representative photographs of migration of A549 cells after treatment with Scaffolds. C, Quantitative statistics of the scratch gaps of A549 cells corresponding to B.


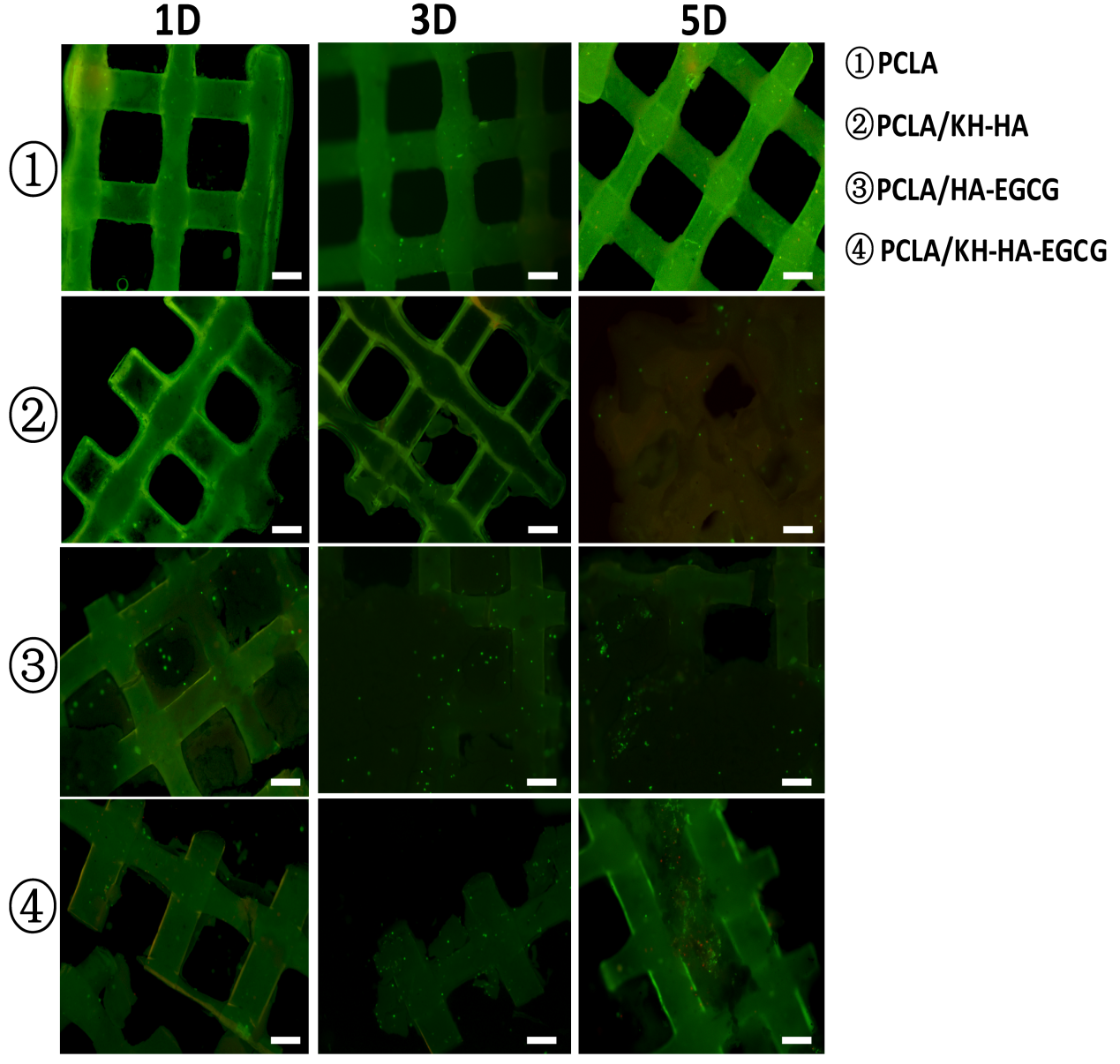


**Figure S7.** Fluorescence microscope images show that the cells of different monolayer scaffold samples adhered at 1, 3, and 5 days. Sacle bar (50μm).

**
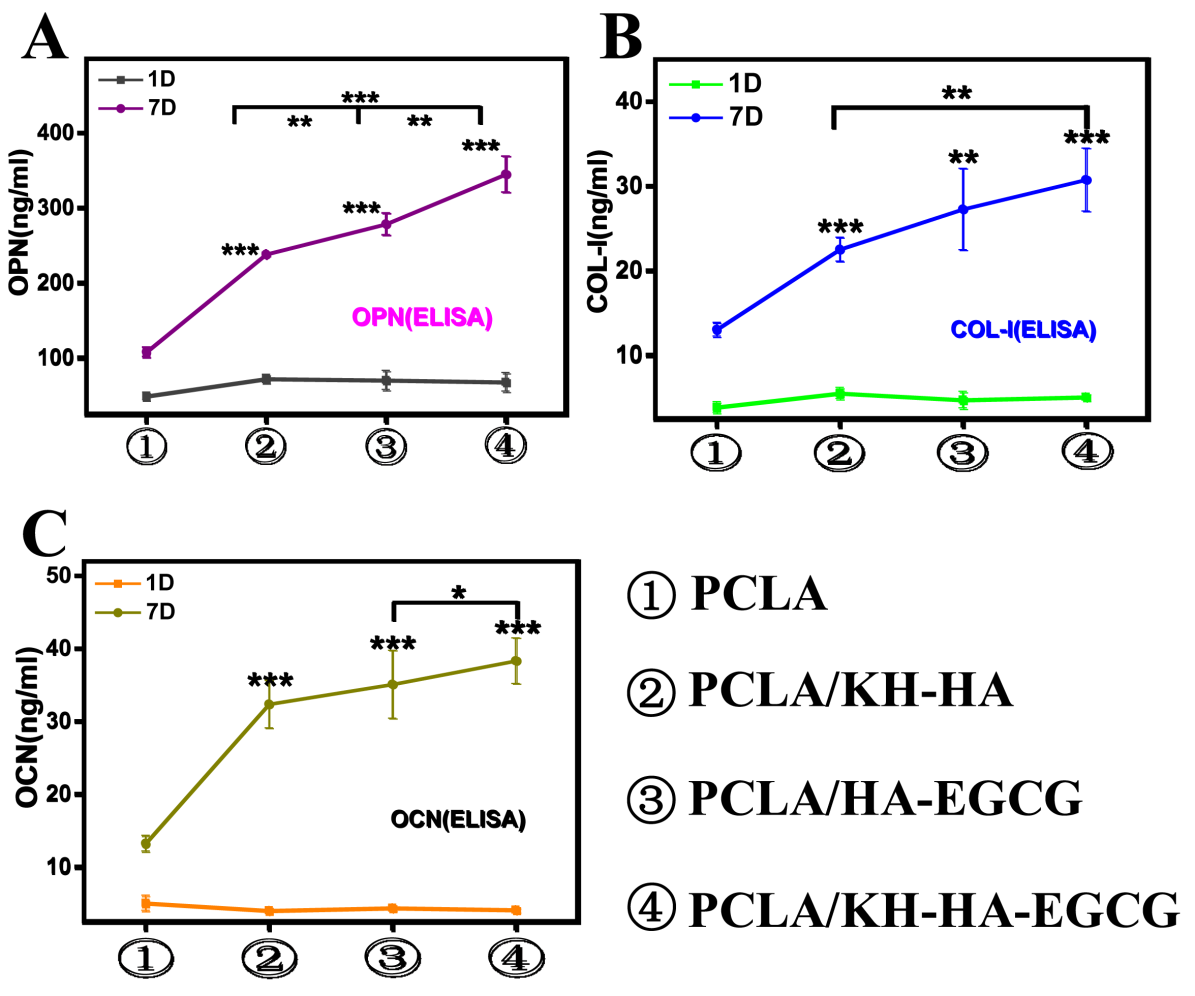
**

**Figure S8.** The effect of different scaffold groups on osteopontin (OPN) protein expression. B, The effect of different scaffold groups on type I collagen (COL-I) protein expression. C, The effect of different scaffold groups on osteocalcin (OCN) protein expression.

**Table S1.** RT-PCR detection primer

| Gene | Forward | Reverse |
| --- | --- | --- |
| BMP-2 | 5'-GGGACCCGCTGTCTTCTAGT-3' | 5'-TCAACTCAAATTCGCTGAGGAC-3' |
| OCN | 5'-GCTCACTCTGCTGACCCTG -3' | 5'-GGGACTGAGGCTCCAAGGTA -3' |
| OPN | 5'-GAGCGGGTGCTTGACAGAC -3' | 5'-GAGCGGGTGCTTGACAGAC-3' |
| ALPL | 5'-CCAACTCTTTTGTGCCAGAGA-3' | 5'-GGCTACATTGGTGTTGAGCTTTT-3' |
| RUNX-2 | 5'-GACTGTGGTTACCGTCATGGC -3' | 5'-ACTTGGTTTTTCATAACAGCGGA -3' |
| COL-IA1 | 5'-TCGTGCCTAGCAACATGCC-3' | 5'-ATTGGGGACCCTTAGGCCAT-3' |
